# Polarization Control in Integrated Silicon Waveguides Using Semiconductor Nanowires

**DOI:** 10.3390/nano12142438

**Published:** 2022-07-16

**Authors:** Ali Emre Kaplan, Valerio Vitali, Valeria Demontis, Francesco Rossella, Andrea Fontana, Samuele Cornia, Periklis Petropoulos, Vittorio Bellani, Cosimo Lacava, Ilaria Cristiani

**Affiliations:** 1 Photonics Research Group, Department of Electrical, Computer and Biomedical Engineering, University of Pavia, 27100 Pavia, Italy; ilaria.cristiani@unipv.it; 2 Istituto Nazionale Fisica Nucleare (INFN)-Pavia Section, 27100 Pavia, Italy; andrea.fontana@pv.infn.it (A.F.); samuele.cornia@unimore.it (S.C.); vittorio.bellani@unipv.it (V.B.); 3 Optoelectronics Research Centre, University of Southampton, Southampton SO17 1BJ, UK; v.vitali@soton.ac.uk (V.V.); pp@orc.soton.ac.uk (P.P.); 4 NEST, Scuola Normale Superiore, Istituto Nanoscienze-CNR, 56127 Pisa, Italy; valeria.demontis@sns.it; 5 Dipartimento di Scienze Fisiche, Informatiche e Matematiche, Università di Modena e Reggio Emilia, 41125 Modena, Italy; f.rossella@unimore.it; 6 Department of Physics, University of Pavia, 27100 Pavia, Italy

**Keywords:** integrated photonics, polarization control, silicon photonics, nanowires

## Abstract

In this work, we show the design of a silicon photonic-based polarization converting device based on the integration of semiconduction InP nanowires on the silicon photonic platform. We present a comprehensive numerical analysis showing that full polarization conversion (from quasi-TE modes to quasi-TM modes, and vice versa) can be achieved in devices exhibiting small footprints (total device lengths below 20 µm) with minimal power loss (<2 dB). The approach described in this work can pave the way to the realization of complex and re-configurable photonic processors based on the manipulation of the state of polarization of guided light beams.

## 1. Introduction

In recent years, the rapid development of integrated photonic circuits has enabled the realization of complex devices serving a variety of technological fields [1,2]. Photonic integrated circuit (PIC) architectures have reached a high level of complexity and can perform several functions, such as the manipulation of optical signals [3,4], modulation at high speed rates [5,6] and quantum and computing operations [7,8]. One of the key functionalities that needs to be further developed is related to the precise and dynamic control of the state of polarization of optical beams traveling within integrated waveguides. It is well known that PIC performance is strongly dependent on the state of polarization of the optical signals. Integrated waveguides are intrinsically asymmetric and characterized by high birefringence, which is even more critical in high-index-contrast integrated platforms [9,10,11]. In addition, most of the active devices for signal processing and modulation need a specific input state of polarization of the signal. To obtain polarization-independent PICs, either the structures should be intrinsically polarization insensitive [12] or a polarization diversity strategy, based on polarization converters and splitters, must be adopted [13]. The latter approach is preferred, since it can be applied to most of the developed PIC architectures that utilize a variety of waveguide dimensions and geometries, enabling advanced design strategies for enabling control over the dispersive and modal properties of devices. As a consequence, the capability to dynamically manage and convert the state of polarization of the light in PICs is key to ensuring polarization-insensitive operations. Polarization converters are needed to select and rotate the state of polarization of a guided mode and place it typically at the quasi-Transverse Electric (TE) or quasi-Transverse Magnetic (TM) state. To date, there have been various demonstrations on polarization manipulation in the literature, for instance, based on the asymmetric geometry of waveguides [14], tapered waveguides [15], metamaterial and periodic structures [16] and other structures [17,18,19]. These approaches typically require relatively complex fabrication processes, and they have poor fabrication tolerances. In addition, they do not allow for post-fabrication trimming or reconfiguration, thereby posing possible restrictions on future developments of novel functionalities, based on circuits on which polarization management and manipulation can be done after fabrication.

**Figure 1:**
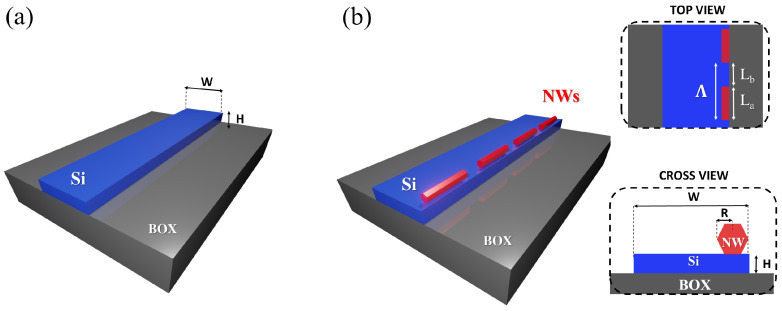
(**a**) Three-dimensional (3D) illustration of a silicon on oxide waveguide structure. (**b**) The same waveguide reported in (**a**) is represented with the addition of NWs. In the right panel, we illustrate the device with additional 2D top and cross views.

In this context, advanced approaches that could provide the tools to control the state of polarization in silicon waveguides, are strongly desired. Semiconductor nanowires (NWs) have emerged, in the last few years, as a powerful class of nanomaterials with notable potential for applications in several different fields including electronics [20,21], photonics [22,23,24,25,26], energy [27,28,29], and sensing [30,31] because of their unique electronic and optical properties and scalable bottom–up growth process [32,33,34]. NWs and NW arrays can be designed and engineered to perform as nanostructured metamaterials and meta-surfaces, artificially prepared electromagnetic materials made of resonant subwavelength structures, showing effective properties that cannot be found in nature. For example, NW metamaterials exhibiting negative refraction, optical cloaks, electrically tunable metamaterials and near-zero reflectance materials have been reported [33,35,36,37]. Advances in nanofabrication and the control of the NWs growth processes have enabled the development of deterministic growth protocols, in which NWs can be grown at selected locations on the substrate with the required size and aspect ratios. Advances in integration between NWs and integrated optical platforms have also been achieved using, for example, monolithic integration and growth approaches [38,39,40]. Although challenging, this quest resulted in notable scientific and technological results [37], and NW fabrication technologies are reaching a sufficient level of maturity to provide clear grounds for their realistic adoption in large-scale integrated systems and in photonic integrated components [33,34,41]. The adoption of NWs or NW arrays on integrated waveguides has been proposed only recently [42], aiming at inducing advanced optical responses in optical waveguides. Here, we propose to adopt NWs and NW arrays to control the polarization of propagating light beams in standard silicon waveguides. The adoption of NWs is envisioned to induce a change in the waveguiding structure, thus in turn allowing to control the state of polarization of an incoming light beam. Our previous results clearly indicate a polarization control effect induced by III–V semiconductor NW arrays—both ordered or disordered [43,44,45]. Indium phosphide was selected among other semiconductors because it was already envisioned as a key material for integration on silicon photonic platforms, as several devices with many optical functionalities such as lasers, detectors, quantum emitters, and modulators were already demonstrated [46]. In this work, we demonstrate through numerical simulations that the state of polarization of light in standard aspect ratio silicon waveguides can be controlled and manipulated by exploiting periodic perturbations induced in the system by the insertion of an array of wurtzite InP NWs (as shown in Figure 1).

The interactions between the optical signal and the NW array have the ability to couple optical power from a TE to a TM guided mode or vice versa with simulated efficiencies close to 100% and insertion losses of the order of a few dB. The proposed approach has a distinctive advantage: NWs are fabricated independently and can be placed on the top of any photonic circuit (with no cladding) during the post-fabrication processes. Therefore, there is no impact on the integrated circuit fabrication routines, and trimming is always possible via precise NW placing using techniques already available and demonstrated in the literature [47,48,49]. Such an approach can be easily extended to several 2D materials that can be deposited on top of optical waveguides to induce a periodic perturbation, thus opening the path to hybrid and reconfigurable integrated platforms enabling polarization processing.

## 2. InP Semiconductor Nanowires

InP nanowires are commonly grown by using bottom–up self assembly techniques and relying on two main processes [34,50]: the catalyst-assisted growth (based on vapor–liquid– solid (VLS) processes, when the catalysts is liquid, or on vapor–solid (VS) processes, when the catalyst is solid) and the catalyst-free growth, which are usually combined with selective area epitaxy [51]. VLS growth is the most commonly adopted process, and it usually relies on the use of metal nanoparticles as catalysts, gold being the most commonly employed metal [52]. The process exploits the eutectic reaction between metal catalyst nanoparticles and the semiconductor source materials in their vapor phase. In the presence of the semiconductor source and above the eutectic temperature for the target metal– semiconductor system, the metal nanoparticle and the semiconductor vapor source forms a liquid metal–semiconductor eutectic alloy droplet, and the system continues to incorporate the semiconductor material until supersaturation. Upon the supersaturation of the liquid alloy, the semiconductor starts to nucleate and precipitate at the liquid–substrate interface beneath the catalyst, giving rise to the nanowire growth [32]. The growth proceeds as long as the continuous transport of precursor components from the gas phase is ensured, and the size and the location of the nanowire are mainly determined by the size and location of the metal nanoparticle used as a catalyst [53]. Catalyst nanoparticles made of one of the elements composing the grown nanowire, rather than metals, can also be employed, the process being in this case named “self-assisted growth” [32]. In catalyst-free growth, the mechanism governing the growth is slightly different, as it is not mediated by any metal or intermediate phase but rather relies on crystal growth rate anisotropies to drive the preferential growth in one dimension [54]. For all the mentioned processes, the most effective approach for obtaining a very fine control over the size, shape and material composition of each individual NWs in an array, as extensively described in [33], is the employment of pre-patterned substrates, realized by nano-lithography techniques, as the growth substrates. The pre-patterned substrates can consist of an array of lithographically defined metal nanoparticles on the bare substrate (in the case of metal-catalyzed VLS growth) or in a pattern of nanoholes inside a thin amorphous oxide mask deposited on the substrate. In the latter case, the process is often called selective area growth (SAG), and it can be combined with both catalyst-assisted and catalyst-free growth. The size and shape of the metal catalyst and of the nanoholes in the dielectric masks represent the key parameters in determining the properties of the grown nanowires.

## 3. Theoretical Background, Device Configuration and Design Strategy

**Figure 2:**
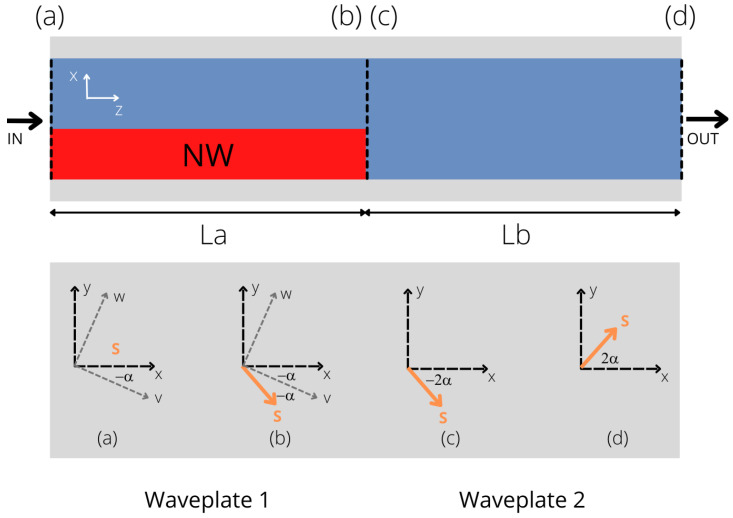
Schematic representation of polarization rotation via halfwave plate asymmetric waveguide subsequent operations. x, y are the waveguide principal axis when no asymmetry is introduced; v, w represent the waveguide axis when the NW is placed; S is the polarization of a linearly polarized propagating beam.

It is well known that the state of polarization of optical beams propagating in planar waveguides can be manipulated via introducing perturbations along the waveguide structure able to break the waveguide symmetry [16]. This results in a rotation of the effective principal axis of birefringence of the waveguide, thus inducing a power coupling between the orthogonally polarized modes. A coherent power transfer from one mode to the orthogonal one can take place when a phase-matching condition is satisfied. This mechanism can be modeled as follows: each waveguide section (with or without perturbation) can operate as a half-wave plate, provided that its length is properly chosen. When the perturbation is present, the principal axes of birefringence are rotated with respect to those of the unperturbed waveguide of an angle α. If we suppose that the polarization at the waveguide beginning is linear, at the output of each half wave-plate section, the polarization will rotate of an angle 2α with respect to its original alignment. Hence, by properly choosing the number of sections, it is possible to rotate the state of polarization of any arbitrary linearly polarized traveling beam. This theoretical picture can be transferred to practice, in planar waveguides, in different ways: one can pattern the waveguide with slanted surfaces or with periodic holes for example [37]. All these implementations are strongly influenced by fabrication imperfections that are very hard to correct during the post-fabrication processes. In this work, we propose to implement such an approach by using InP NWs. Specifically, we envision to reproduce two consecutive half-wave plate sections, one realized with the insertion of the NW and the other one, without the NW, as depicted in Figure 2. The insertion of an NW induces an asymmetrical perturbation in the propagation constants that results in the excitation of quasi-TE (TE${}^{\prime }$) and quasi-TM (TM${}^{\prime }$) modes that are rotated with respect to the principal axes of the waveguide by an angle α that depends on the NW position and size. If we indicate with $L_{a}$ and $L_{b}$ the lengths of the sections with and without the NW, respectively, the phase-matching condition to ensure the equivalent (obtained on planar waveguides) effect of half-wave plates can be expressed by the following equations:

(1) $$L_{a}=\frac{\lambda }{2(n_{{\mathrm{TE}}^{{}^{\prime }}}-n_{{\mathrm{TM}}^{{}^{\prime }}})}$$

(2) $$L_{b}=\frac{\lambda }{2(n_{\mathrm{TE}}-n_{\mathrm{TM}})}$$

where λ is the operating wavelength, $n_{{\mathrm{TE}}^{{}^{\prime }}}$, $n_{{\mathrm{TM}}^{{}^{\prime }}}$ are the effective mode indexes of the TE${}^{\prime }$ and TM${}^{\prime }$ modes calculated for the section with the NW, while $n_{\mathrm{TE}}$, $n_{\mathrm{TM}}$ express the effective mode indexes of the TE and TM modes without NW. The period Λ of the perturbation is given by $\Lambda =L_{a}+L_{b}$. At the end of each period composed of two halfwave-plates, the polarization of the beam *S* is rotated (counterclockwise in Figure 2) by an angle 2α. The value of the angle α is a very critical parameter; for instance, if α=π/4, one waveplate ($L_{a}$) section would allow converting a linear polarized TE beam to a linearly polarized TM beam. When α<π/4, a periodic structure is needed, ensuring that full coupling is achieved after a few periods. In order to verify the feasibility of such a set-up and study the influence, on the device performance, of the various parameters that characterize the structure, we performed a full numerical campaign, using a 3D commercial Lumerical Finite Difference Time Domain (FDTD) package. The numerical set-up was composed of a standard silicon waveguide (size W = 450 nm, H = 220 nm) where a periodic perturbation with period Λ is introduced by placing an array of InP NWs (refractive index *n* = 3.56) in an asymmetric position (i.e., at the border of the waveguide) on the waveguide top. A non-uniform mesh setting was employed in the FDTD simulation, where the minimum mesh size utilized had a size of 5 nm (with mesh with higher resolution being positioned around the NW regions). The input signal is a linearly polarized TE waveguide optical mode in the wavelength range of the C-band around λ = 1550 nm.

## 4. Numerical Results

**Figure 3:**
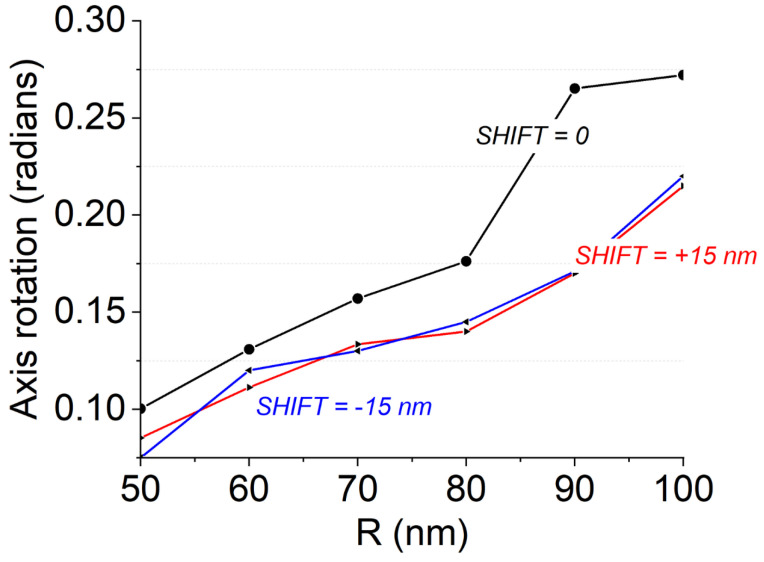
Optical axis rotation introduced by the addition of a NW to a standard waveguide to break the device symmetry; the blue and red curves show results obtained by shifting the horizontal position of NWs.

As a first step of our numerical analysis, we evaluated the efficiency of the insertion of a single NW in breaking the waveguide symmetry. This can be quantified by evaluating the rotation of the principal optical birefringence axis as a function of the NW parameters (size, *R*, and NW placing position), namely the parameter α according to the schematic presented in Figure 2. Intuitively, the position at which the symmetry-breaking effect is maximized is the one depicted in Figure 1, in which the NW is placed at one edge of the waveguide (the bottom right-hand vertex of the hexagon positioned at the right-hand edge point of the waveguide; the same effect can be obtained by placing the NW at the left section). We adopted this configuration, and we calculated α as a function of the NW size (*R*); results are reported in Figure 3. It can be observed that the rotation angle cannot be larger than α=π/10 that is obtained when *R* = 100 nm, which is the largest size allowed by our fabrication capabilities. To verify that the position selected in this simulation stage is indeed the one that maximizes the symmetry-breaking behavior, we reproduced the curve shown in Figure 3 when the NWs were horizontally shifted by ±15 nm with respect to the original position depicted in Figure 1: all the configurations with non-zero shifts were characterized by lower α angles for any *R* NW configuration, thus verifying our initial assumption.

**Figure 4:**
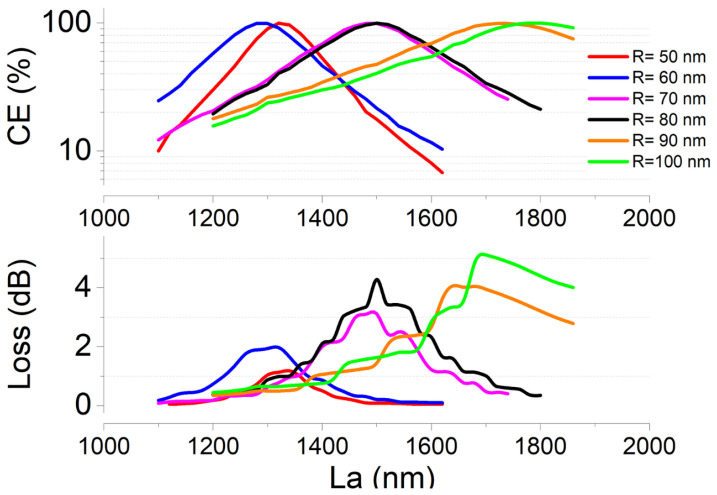
Top panel: CE calculated as a function of the NW radius. The number of NWs is assumed to be the minimum to achieve the maximum conversion. Note that the scale is not linear. Bottom panel: associated total losses calculated as the ratio between the output power and the input power levels.

**Figure 5:**
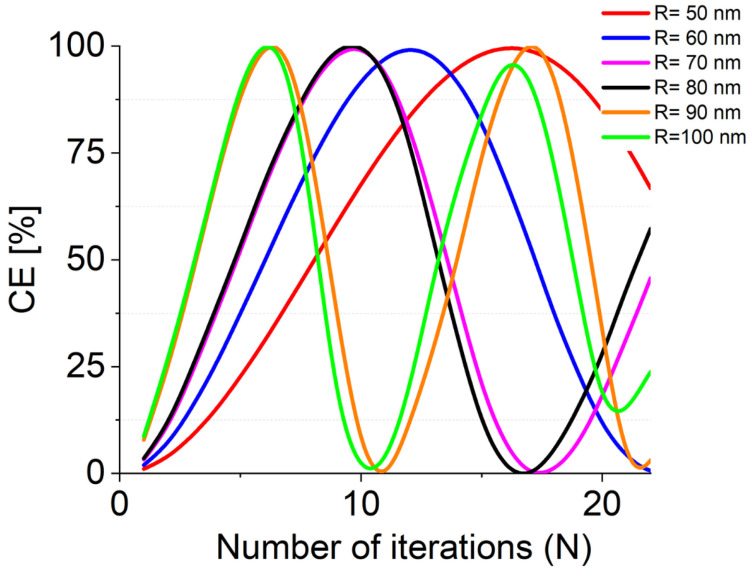
Calculated CE as a function of N, number of iterations required to achieve full TE–TM conversion.

The second step of the numerical campaign was focused on scanning the various NWs InP parameters, aiming at finding the best device configuration minimizing the length L of the polarization conversion device (L= $(L_{a}+L_{b})N$ with *N* being the number of iterations required to achieve full TE–TM conversion) and exhibiting the lowest possible total loss value. To pursue this strategy, we first calculated the TE and TM modal distributions (and consequently $n_{\mathrm{TE}}$ and $n_{\mathrm{TM}}$) for the standard waveguide, without the NW, depicted in Figure 1, for a central λ=1550 nm. These results allowed calculating the length of the sections without NW, namely $L_{b}$ (see Figure 2) that was determined to be 1.248 µm. Subsequently, we calculated the polarization conversion efficiency (CE) defined as

(3) $$\mathrm{CE}=\frac{P_{\mathrm{TM}}}{P_{\mathrm{TE}}+P_{\mathrm{TM}}}\times 100$$

where $P_{\mathrm{TM}}$ and $P_{\mathrm{TE}}$ are the power measured for each polarization at the end of the device under test, the total device losses and the number of the required iterations to attain complete TE–TM conversion via scanning the NW’s free parameter, namely *R* and $L_{a}$, for various wavelength values. Results of the numerical campaign are reported in Figure 4 and Figure 5.

In Figure 4 in the top and bottom panels, we observe that full CE can be achieved for the whole set of *R* values considered. The exploitation of NWs with larger *R* require longer $L_{a}$ sections: this can be explained by the fact that larger *R* values reduce the waveguide asymmetry, that, as a consequence, results in less birefringent. Larger *R* values are, however, also accompanied by higher values of losses. Indeed, as expected, the addition of NWs with a hexagonal shape, exhibiting a refractive index that is comparable to that of the waveguiding material (3.56 compared to 3.44 showed by Si), originates an additional loss mechanism due to the scattering that the propagating light experiences at the waveguide–NW edges. This effect can be observed in Figure 4 (bottom) where a minimum loss of 1.5 dB is achieved for the waveguide loaded by NWs with *R* = 50 nm while, in contrast, losses up to 5 dB are found for waveguides loaded by NWs with R> 80 nm.

**Figure 6:**
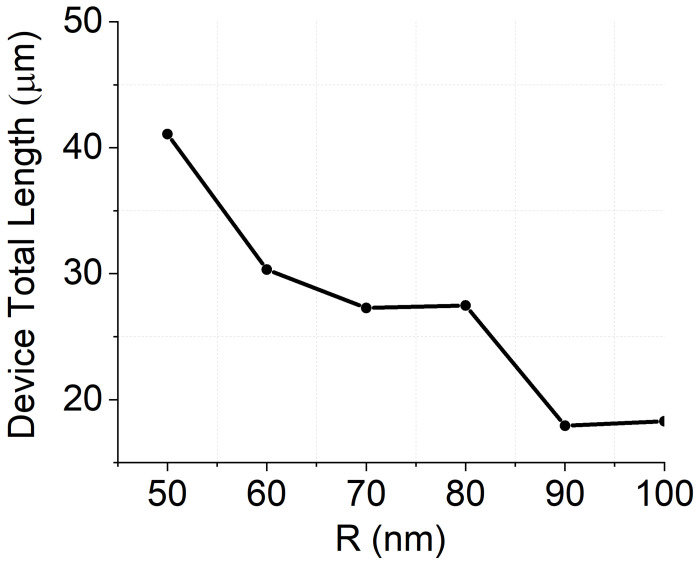
Calculated device total length for different *R* configurations.

The number *N* of iterations required to achieve full conversion (or intermediate conversion values) are reported in Figure 6 as a function of *R*. Here, we note that smaller values of *R* require a larger number of periods in accordance with the theoretical background (see Figure 2). In particular, $L_{T}$ = 42 µm is required to achieve full conversion for *R* = 50 nm, while $L_{T}$ = 19 µm is sufficient if *R* = 90 nm.

**Figure 7:**
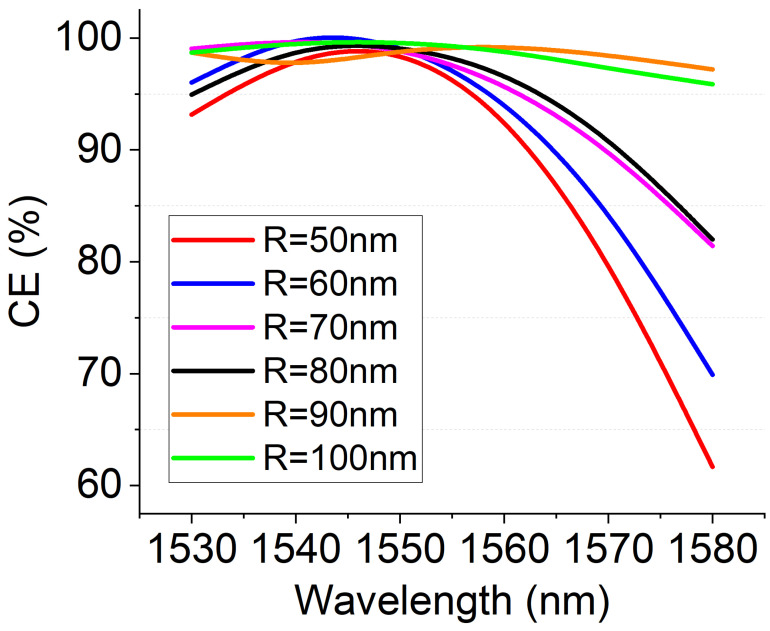
CE conversion bandwidth as a function of R. CE is calculated as the ratio between the optical power at the output port allocated on the TM mode over the total power.

Another critical aspect of the polarization conversion function device is the operational bandwidth. It is clear that the phase-matching condition is fully satisfied at a specific wavelength and the bandwidth depends both on the number *N* of periods and the dispersive behavior of the waveguide. We calculated the bandwidth response for each configuration previously analyzed. Results are shown in Figure 7. As expected, polarization converters with a low number of NWs employed (N< 6) show a bandwidth response with a 90% conversion bandwidth that exceeds the C-band (BW > 80 nm). The bandwidth becomes significantly lower when R< 70 nm, which is a configuration that is set to require a larger number of NWs (N> 10).

We also note that for NWs with a length below 3 µm, the random diameter variations which can occur in real bottom–up grown nanowire samples were estimated to be below 5%, with negligible NW variations along the NW axis [55,56]. According to our results, these variations are not expected to substantially affect the device performances.

## 5. Conclusions

In this work, we proposed a novel approach to realize polarization converters in silicon photonic-based platforms. Specifically, we proposed to integrate semiconductor NWs into the platform, aiming at breaking the waveguide symmetry and, therefore, introducing a mechanism that allows coupling light from one polarization to another. We performed a full numerical campaign based on a standard silicon waveguide integrated with InP NWs. We showed that full conversion between linear polarization (TE–TM) can be achieved when the NWs size are of the order of 50–100 nm in terms of radius. We also analyzed the impact of the NW fabrication and positioning parameters, thus providing a comprehensive tool to design a real small footprint polarization converter device.

## Data Availability

Data can be provided after request to corresponding authors.
